# On phase transitions to interdisciplinary and convergent research

**DOI:** 10.1093/pnasnexus/pgag219

**Published:** 2026-07-01

**Authors:** Yannis C Yortsos

**Affiliations:** Editor-in-Chief, PNAS Nexus, USC Viterbi School of Engineering, University of Southern California, Los Angeles, CA 90089-1450, USA

This spring marked 4 years since the first articles were published in *PNAS Nexus*, with submissions to the journal starting in August 2021. I have had the privilege of serving as its Editor-in-Chief since December 2022. Today, thanks to the remarkable work of our Deputy and Associate Editors and our Board of Reviewing Editors, the journal is solidifying its position as the home of exceptional interdisciplinary and convergent research, as well as high-quality disciplinary research. Since August 2021, we have received nearly 6,000 submissions and published more than 1,800 articles covering a wide breadth of disciplines. To handle the breadth and volume of submissions, an editorial board of more than 130 experts volunteers their time and expertise to help provide a thoughtful, inspiring, and welcoming open access home.

**Figure:**
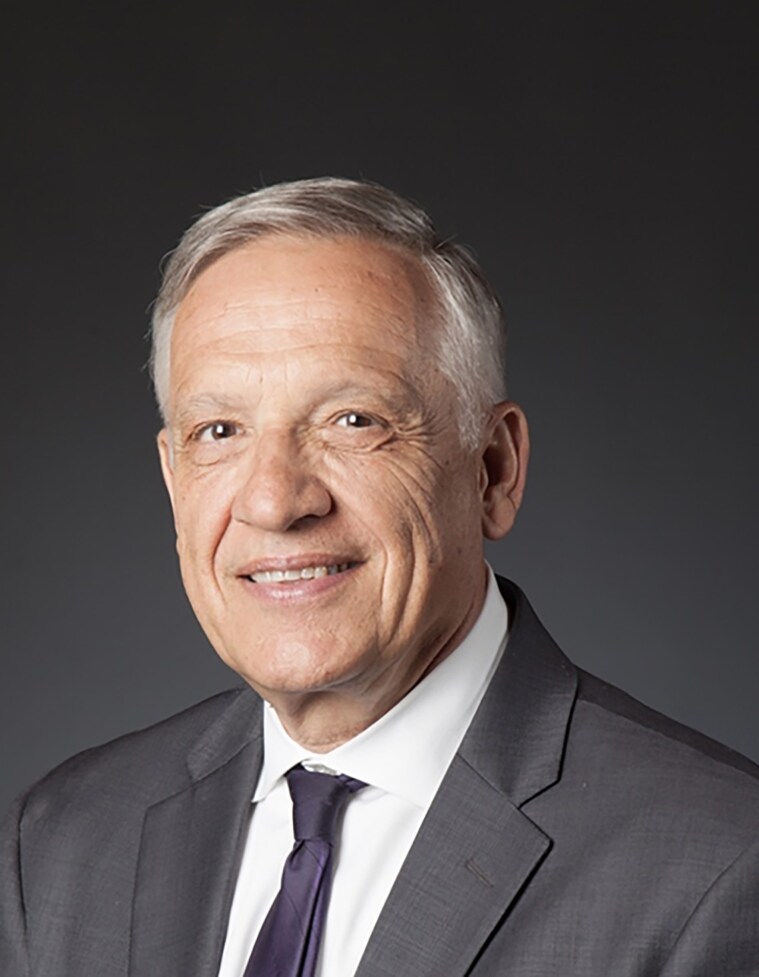
Yannis C. Yortsos

Looking across recent *PNAS Nexus* articles, we can see that their strongest commonalities are structural and conceptual, as expected for a journal spanning many disciplines. Unifying themes include Bridging Scales (from micro to macro), Complex Systems Thinking (from networks to nonlinear dynamics), Human–Technology Interactions (including AI and LLMs, technical innovations, and social consequences), Societal Relevance (linking foundational research to real-world challenges, and how computational systems interact with human cognition, norms, and institutions), and Methodological Innovation across the disciplines (connecting mechanisms to outcomes). Now, and while I must disclose that the above synthesis was aided by ChatGPT, the themes identified align closely with our own editorial experience.

In my last editorial of October 2025, I suggested that AI will help accelerate research convergence and interdisciplinarity, a natural home for which will be *PNAS Nexus*. We already see fast-increasing evidence of this penetration. Many in the popular media have suggested that we are entering a new phase, that we are undergoing a phase transition to the age of AI. Indeed, phase transition is not an uncommon term in describing strong innovation processes, such as AI. Be that as it may, however, my inner chemical engineer cannot let this use of physical phenomena language go unchallenged.

So, here goes: Let *A* denote a measure of innovation, and assume that its rate of change follows a power law, namely $dA/dt=kA^{m}$, where *k* is a kinetic constant, and m>0 is the power-law exponent. (A note that one could devise an auto-catalytic chemical reaction that leads to such a rate expression, with m=1 being a first-order reaction, etc.) Now, m=1 leads to an exponential, A∼exp(kt), mimicking “Moore's law.” Such innovation is unlikely to lead to a sharp phase change (although such would be notable over historical periods). However, if m>1, the resulting solution is $A∼(t^{*}-t{)}^{1-m}$, suggesting a “singularity” at $t^{*}$. Technological “singularities” were suggested as early as 2005 by Kurzweil (1). Is it possible that the extraordinary pace of change we are witnessing today is closer to such a singular behavior? Selecting as a measure of change the number of data used to train LLMs as a function of time, one can show that such a possibility is indeed real. Which brings me to my final conjecture regarding a phase transition, and which relies on drawing a parallel of *A* with a “correlation length.” If innovation approaches a singular behavior, so would the associated correlation length, whose behavior in physics signals the onset of a phase transition.

The casual reader of this short essay would, of course, not need this elaborate exercise to accept that we are entering a new phase. And while a more rigorous reader might find the above acrobatics a bit contrived, unquestionably, we are in a new phase of discovery and convergence in the science and technology endeavors, with a potentially extraordinary impact. It is our goal at *PNAS Nexus* to be a premier journal where such discovery is disseminated and in which the deep domain knowledge of the authors of its interdisciplinary papers will help obtain from the underlying interdisciplinary (and convergent) research new building blocks that will themselves augment their disciplinary domains in an unceasing quest for higher perfection.

As I close this editorial, I would be remiss not to mention our latest impact factor of 4.8. As we build upon a successful 4 years, I should mention our future goals and direction for the sustained growth of the journal. We are focused on improving communication with authors and strengthening the journal's editorial processes, as well as expanding our commissioning activities with Calls for Papers in emerging research areas. As we continue to build the foundational knowledge in a new phase of discovery and convergence, we hope the impact of *PNAS Nexus* continues to rise, reflecting the quality and rigor of the work done. Please join us on our journey.
